# The Retina as a Proxy for Brain Neurodegeneration: A Narrative Review on OCT-Based Retinal Imaging in the Early Detection of Alzheimer’s and Parkinson’s Disease

**DOI:** 10.3390/jimaging12030104

**Published:** 2026-02-27

**Authors:** Ouafa Sijilmassi

**Affiliations:** Optics Department, Faculty of Optics and Optometry, Complutense University of Madrid, C/Arcos de Jalón, 118, 28037 Madrid, Spain; o.sijilmassi@ucm.es

**Keywords:** retinal imaging, optical coherence tomography, Alzheimer’s disease, Parkinson’s disease, neurodegeneration, biomarkers, review

## Abstract

Neurodegenerative diseases, including Alzheimer’s disease (AD) and Parkinson’s disease (PD), are major causes of cognitive and motor decline, yet early diagnosis remains challenging due to asymptomatic phases and limited non-invasive biomarkers. This narrative review systematically synthesized studies on retinal imaging in AD and PD. Published studies were identified through searches of PubMed, MEDLINE, Google Scholar, and reference lists, focusing on Optical Coherence Tomography (OCT), OCT Angiography (OCTA), and Spectral-Domain OCT (SD-OCT) assessing retinal structural and vascular changes. Data were extracted on retinal layer thickness, vascular parameters, and diagnostic metrics. Findings indicate that both diseases consistently exhibit thinning of inner retinal layers, particularly the retinal nerve fiber layer (RNFL) and ganglion cell–inner plexiform layer (GCIPL). In AD, studies reported progressive inner retinal thinning across disease stages, sometimes accompanied by outer retinal and retinal pigment epithelium changes. In PD, thinning was observed predominantly in RNFL and GCIPL, correlating with disease duration and motor severity. Microvascular alterations were described in both disorders, with disease-specific spatial patterns reported across studies. Overall, retinal imaging emerges as a non-invasive, high-resolution, and cost-effective tool for early detection, differential assessment, and longitudinal monitoring of neurodegenerative diseases. These findings support the translation of retinal biomarkers into clinical practice for improved disease management.

## 1. Introduction

Neurodegenerative diseases (NDs) are among the leading causes of disability and mortality worldwide and represent a growing public health concern due to their increasing prevalence in aging populations [1]. These disorders are characterized by progressive neuronal loss, leading to functional impairment and brain atrophy. Among the most prevalent neurodegenerative conditions are Alzheimer’s disease (AD) and Parkinson’s disease (PD).

AD is the most common neurodegenerative disorder, accounting for approximately 60–80% of all dementia cases. It primarily affects brain regions involved in memory, language, and cognitive processing, which explains why early clinical manifestations typically include deficits in memory, communication, and executive function [2].

PD, in contrast, is a clinical syndrome of parkinsonism associated with the degeneration of pigmented nuclei in the brainstem. As the second most frequent neurodegenerative disorder after AD, PD is a chronic and disabling condition that substantially compromises motor function and quality of life [3].

In addition to these clinical manifestations, neurodegenerative diseases impose substantial emotional, physical, and economic burdens on patients, caregivers, and healthcare systems. As AD and related dementias progress, affected individuals require increasing levels of care, including medical treatment, assistive devices, home adaptations, and ultimately long-term residential support. These escalating needs place considerable financial strain on families and society, with indirect costs largely driven by caregiver time, lost income, and reduced productivity. Moreover, many neurodegenerative disorders have a prolonged asymptomatic phase, during which pathological changes precede overt cognitive or motor symptoms, contributing to delayed diagnosis. Although current therapies aim to slow disease progression and improve quality of life, disease-modifying treatments remain limited [4,5,6,7].

The eye represents the only anatomical site in the body where neuronal structures and vascular networks can be directly visualized in vivo. Beyond this unique accessibility, the eyes and their neural connections to the brain constitute the visual system, a highly organized network responsible for transmitting sensory information from the external environment to central visual processing centers. This process relies on precisely regulated axon guidance mechanisms that enable retinal ganglion cell axons, the only retinal neurons projecting beyond the eye, to reach and synapse with their target regions in the brain [8]. Owing to its complex cellular architecture and direct anatomical and functional connection to the brain, the retina represents a unique and accessible extension of the central nervous system (CNS), offering a window into neural processes that are otherwise difficult to study in vivo [9].

In this context, the retina has emerged as a promising tool for the early detection and monitoring of neurodegenerative diseases, which is critical for improving clinical management and facilitating the development of disease-modifying therapies [10]. Retinal imaging techniques can reveal structural and functional alterations associated with disorders such as AD and PD, supporting their use as noninvasive biomarkers of neurodegeneration. Compared with traditional tissue- or fluid-based biomarkers, retinal imaging provides diagnostic and prognostic information in a less invasive and more cost-effective manner, highlighting its growing translational relevance [11].

Despite advances in retinal imaging and growing evidence of retinal alterations in neurodegenerative diseases, a comprehensive synthesis of findings from OCT, OCTA, and SD-OCT studies across both AD and PD remains limited. A clear understanding of structural and vascular retinal changes, their disease-specific patterns, and potential clinical applications is essential for translating retinal biomarkers into practice. Therefore, this review was conducted to systematically summarize current evidence, compare retinal imaging findings between AD and PD, and highlight their potential role in early detection and disease monitoring.

This review summarizes the current state of retinal imaging biomarkers in neurodegenerative diseases, with particular emphasis on AD and PD, and discusses their potential clinical applications.

## 2. Methods

### 2.1. Search Strategy

Published studies were identified through systematic searches of PubMed, MEDLINE, Google Scholar, and other relevant databases for human studies published up to the date of this review. Search terms included combinations of “retina,” “retinal nerve fiber layer,” “ganglion cell layer,” “macula,” “optical coherence tomography,” “spectral-domain OCT,” “optical coherence tomography angiography,” “Alzheimer’s disease,” “Parkinson’s disease,” “neurodegeneration,” and “cognition.” Titles and abstracts were screened to identify studies focusing on OCT and OCTA imaging in AD and PD. Additionally, the reference lists of all included articles were hand-searched for further relevant studies.

### 2.2. Inclusion and Exclusion Criteria

Studies were included if they met the following criteria:

- Original research article.
- Written in English.
- Used OCT or OCTA (including SD-OCT) to assess retinal structure or microvascular changes.
- Included patients diagnosed with AD and PD according to well-established clinical criteria.

Exclusion criteria were:

- Case reports or conference abstracts.
- Studies involving animal models.
- Studies not reporting sample size, imaging methodology, or any relevant retinal parameters.
- Studies that used retinal imaging methods other than SD-OCT or OCTA, such as fundus photography, laser Doppler, or other non-OCT-based techniques.

### 2.3. Data Extraction

Studies were screened by title and abstract to assess their relevance. Duplicate and irrelevant studies were excluded. Full texts of remaining studies were then reviewed to confirm eligibility based on the inclusion and exclusion criteria. The following data were extracted from each eligible study:

- First author and year of publication.
- Study aims and objectives.
- Study design and population (number of AD, PD, and control subjects).
- Type of OCT used (e.g., SD-OCT, OCTA).
- Retinal parameters assessed (e.g., circumpapillary retinal nerve fiber layer (cpRNFL), ganglion cell-inner plexiform layer (GC-IPL), macular thickness, vascular density, foveal avascular zone (FAZ)).
- Method of analysis (manual or automated segmentation (AI)).
- Reported diagnostic performance metrics, such as area under the receiver operating characteristic curve (AUC), sensitivity, specificity, and accuracy, where available.

This review focuses on studies reporting retinal structural and vascular changes in AD and PD using OCT-based imaging. The review prioritizes studies that directly address the main outcomes of interest; however, some relevant references outside this scope may not have been captured.

## 3. The Relationship Between the Retina and the CNS

### 3.1. The Retina as a Component of the CNS

The retina and the brain share a common embryological origin, as both arise from the anterior neural tube. During early development, in the third week of gestation, the optic vesicles evaginate from the diencephalon, a forebrain structure that later gives rise to the thalamus, establishing a direct developmental link between the retina and specific brain regions [12]. Functionally, the retina constitutes an integral component of the CNS and communicates with the visual cortex through a well-defined synaptic pathway involving the optic nerve, the lateral geniculate nucleus, and the optic radiations. Accordingly, the retina shares multiple defining features with the brain, including neuronal and glial cell populations, specialized blood–neural barriers, immune privilege, and tightly regulated vascular homeostasis [13]. These shared characteristics underpin the concept of the retina as an accessible surrogate for assessing brain structure and function [14].

Both the retina and the brain are highly metabolically active tissues that rely on dense and specialized vascular networks to meet their substantial energy demands. Although retinal and cerebral vasculature exhibit comparable blood–neural barrier properties and regulatory mechanisms, they are not fully equivalent, as significant differences in molecular composition and functional responses to physiological and pathological stressors have been described [15]. Nevertheless, a strong anatomical correspondence exists between retinal and cerebral circulation at both macrovascular and microvascular levels [16].

In summary, the strong anatomical and functional correspondence between retinal and cerebral tissues, combined with advances in noninvasive retinal imaging, supports the use of the retina as a surrogate for studying neurodegenerative mechanisms in the CNS. Moreover, numerous neurodegenerative diseases are associated with detectable retinal alterations, reinforcing the value of retinal assessment as a noninvasive indicator of brain pathology [17].

### 3.2. Causes and Effects of Neurodegenerative Diseases on the Retina

**Figure 1:**
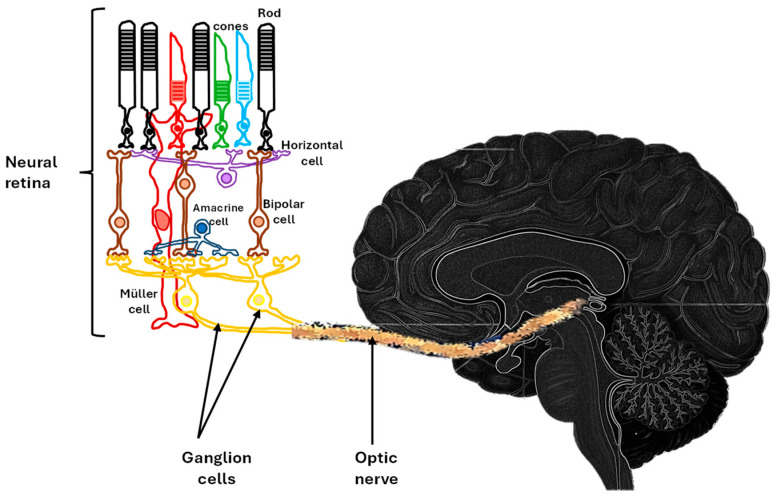
Anatomical relationship between the neural retina and the brain. Schematic representation of the neural retina as an extension of the CNS. Retinal ganglion cells receive visual information from photoreceptors through bipolar and amacrine cells and collectively transmit both image-forming and non-image-forming visual signals via the optic nerve along the visual pathway to the brain. This direct anatomical and functional connection supports the concept of the retina as a reflection of brain structure and function and underlies its use as a non-invasive biomarker of neurodegeneration.

The retina comprises five major neuronal cell types that are essential for its structural organization, visual signal processing, and functional integrity: photoreceptors (rods and cones), horizontal cells, bipolar cells, amacrine cells, and retinal ganglion cells. Under physiological conditions, photoreceptors detect light stimuli and convert them into graded changes in membrane potential. These signals are transmitted to bipolar cells, while horizontal cells modulate lateral signal integration. Retinal ganglion cells receive synaptic input from bipolar and amacrine cells and convey visual information to higher visual centers through their axons in the inner plexiform layer (IPL) [18,19]. Neural retina connects to the brain through the optic nerve (Figure 1).

Beyond neuronal circuits, additional retinal components play a critical role in visual processing and tissue homeostasis. The retinal pigment epithelium (RPE) is responsible for visual pigment regeneration, whereas Müller glial cells provide metabolic support, regulate synaptic remodeling, and secrete neurotrophic factors. The coordinated interaction between retinal neurons and glial cells enables vertical and horizontal signal integration, supporting essential visual functions such as color discrimination, visual acuity, and contrast sensitivity [20].

Impairments in color vision have been associated with cognitive decline and may be detectable during early or prodromal stages of neurodegenerative disease, even in individuals with preserved visual acuity [21,22]. Pathological brain changes may be reflected in the retina as thinning of the retinal nerve fiber layer (RNFL), ganglion cell layer (GCL), and IPL [23,24]. Apoptosis of retinal ganglion cells can lead to anterograde degeneration, resulting in RNFL thinning and, in some cases, reduced gray and white matter volumes in brain regions associated with the visual pathway [25,26,27]. Consistently, Salobrar-García et al. [28] reported both structural and functional retinal alterations in patients with mild and moderate AD, in the absence of ocular comorbidities that could confound the results.

Recent advances in retinal imaging have highlighted microvascular alterations as potential non-invasive biomarkers for neurodegenerative disease detection. Given the shared embryological origin and anatomical similarities between retinal and cerebral vasculature, retinal microvascular changes may reflect CNS vascular pathology. Studies have described vascular abnormalities in the retinas of patients with AD and PD, including increased vessel tortuosity, venous narrowing, reduced retinal blood flow, microvascular network disruption, and decreased vascular branching complexity [29,30].

These neuronal and vascular alterations in the retina underscore the potential of retinal imaging as a window into CNS pathology, motivating the development of structural and functional imaging techniques for early diagnosis.

## 4. Retinal Imaging Techniques for Diagnosing Neurodegenerative Diseases

Diagnosing neurodegenerative disorders remains challenging, particularly due to the difficulty of identifying pathological changes at early stages when therapeutic interventions are most effective. Consequently, there is growing interest in complementary diagnostic tools that extend beyond conventional clinical evaluations. Retinal imaging techniques have emerged as a promising area, as they enable in vivo, high-resolution, and real-time visualization of neural tissue alterations associated with neurodegenerative diseases such as AD and PD.

Several advanced retinal imaging modalities are currently being investigated, including Optical Coherence Tomography (OCT), OCT Angiography (OCTA), and Spectral-Domain OCT (SD-OCT). Each technique provides distinct advantages, offering detailed information on retinal morphology, layer thickness, and microvascular integrity that cannot be obtained through traditional fundus photography or hyperspectral imaging alone. Integration of complementary approaches, such as combining OCT with hyperspectral imaging, has demonstrated considerable potential to improve both accuracy and efficiency in diagnosing neurodegenerative conditions [31].

### 4.1. Optical Coherence Tomography

OCT is a non-invasive optical imaging technique capable of producing cross-sectional images with near-histological resolution and a penetration depth of a few millimeters. This fast and highly reproducible method relies on low-coherence interferometry to analyze backscattered light from biological tissues. Sequential reflectance profiles are acquired as the scanning beam traverses the sample, resulting in two-dimensional images or three-dimensional volumetric reconstructions [32].

In retinal applications, OCT provides detailed structural information on the RNFL, retinal neurons, and vascular components. Axonal loss is commonly assessed through RNFL thickness, whereas neuronal damage is evaluated using measurements of GCL or the combined ganglion cell-inner plexiform layer (GCIPL) [33].

#### OCT Findings in PD and AD: Comparative Discussion

OCT studies show that both PD and AD are associated with measurable retinal neurodegeneration, but the patterns differ.

**Parkinson’s Disease:**

- Retinal thinning predominantly involves peripapillary RNFL (pRNFL) and ganglion cell-related layers, including GCL, GCIPL, ganglion cell complex (GCC), IPL, and inner nuclear layer (INL) [34,35,36,37,38,39].
- Alterations occur even in early or non-demented PD cohorts, suggesting early retinal involvement [34,35,40].
- RNFL and GCC thinning correlate with disease duration, severity, cognitive decline, and visual dysfunction [34,41,42].
- Specific vulnerability is observed in peripapillary regions, sometimes preceding macular involvement [43,44].
- No significant differences were found in the thickness of most of the outer retinal layers compared to healthy controls, suggesting a general preservation of the outer retina in PD [45,46]; however, consistent thinning of the outer nuclear layer (ONL) has been reported across various OCT studies, indicating selective photoreceptor somatic degeneration rather than widespread outer retinal involvement [34,38].

**Alzheimer’s Disease:**

- Thinning of the RNFL, GCC, and GCL–IPL has been consistently reported in AD, with preferential involvement of the temporal and inferior peripapillary RNFL regions, although some inter-study variability remains [47,48,49,50].
- Inner retinal thinning shows significant correlations with cognitive impairment, hippocampal and entorhinal cortex atrophy, and overall disease progression [51,52].
- A comprehensive review by Min et al. [53] synthesizes OCT findings across the AD continuum, from subjective cognitive decline (SCD) and mild cognitive impairment (MCI) to AD dementia, highlighting the consistent vulnerability of inner retinal layers.
- At preclinical stages such as SCD, significant pRNFL thinning has been observed compared with cognitively normal controls, and macular GCC thickness has been shown to correlate positively with cerebral blood flow, suggesting that early retinal neuronal changes may parallel initial cerebral alterations along the AD continuum [47].
- During the MCI stage, diffuse thinning of inner retinal layers is commonly observed, including reductions in macular GCIPL, macular GCC, macular GCL, and macular RNFL [49].
- In patients with established AD, a progressive and consistent pattern of thinning affects the inner macular layers, GCIPL, GCC, macular RNFL, and total macular thickness, accompanied by bilateral pRNFL thinning that correlates positively with cerebral brain volumes [49,50].

In summary: Overall, PD and AD share overlapping OCT phenotypes characterized by inner retinal thinning, particularly involving ganglion cell-related layers. However, PD demonstrates a more uniform and reproducible pattern of retinal degeneration with clearer associations with disease duration and severity, whereas AD exhibits greater heterogeneity, reflecting the complexity of the cognitive impairment continuum. These distinctions suggest that OCT may serve as a general marker of neurodegeneration, while disease-specific patterns and clinical correlations are essential for accurate interpretation. Future longitudinal studies using standardized OCT protocols will be critical to clarify the temporal evolution of retinal changes and to enhance the clinical utility of OCT for differentiating and monitoring neurodegenerative disorders.

### 4.2. Optical Coherence Tomography Angiography

OCTA is a non-invasive imaging modality that uses motion-contrast principles to generate three-dimensional maps of intraocular vascular networks. This technique allows visualization of both retinal and choroidal vasculature without the need for intravenous contrast, providing detailed structural and functional vascular information [54,55].

The motion-contrast signal is derived from pixel-to-pixel intensity variations between consecutive OCT B-scans. Pixels with intensities below a predefined threshold are excluded, producing masked areas in the angiogram. As a result, meaningful OCTA data can only be obtained from regions with sufficiently strong underlying OCT signals [56].

OCTA has demonstrated the ability to detect multiple microvascular alterations, including choriocapillaris abnormalities, retinal microvascular changes such as capillary rarefaction, non-perfusion areas, alterations of the foveal avascular zone (FAZ), vascular remodeling around the FAZ, and increased capillary tortuosity described in various retinal and neurological diseases. Furthermore, OCTA enables visualization of deeper vascular plexuses, which enhances our understanding of the mechanisms underlying vascular pathology. This imaging modality has proven particularly valuable in assessing vascular changes in dry age-related macular degeneration [57], glaucoma [58], and diabetic retinopathy [59], among other conditions.

#### OCTA Findings in PD and AD: Comparative Discussion

OCTA studies have consistently revealed retinal microvascular alterations in both PD and AD or MCI patients.

**Parkinson’s Disease:**

- Early microvascular alterations primarily affect the superficial capillary plexus (SCP) of the macula. Several studies have consistently reported reductions in macular vessel density (VD), perfusion, fractal dimension (FD), and capillary complexity in parafoveal, perifoveal, and total SCP regions, reflecting microvascular remodeling associated with neurodegeneration [44,60,61,62].
- Alterations of the FAZ are also reported, with PD patients exhibiting smaller FAZ areas, decreased circularity, and increased vascular complexity or lacunarity, highlighting localized microvascular remodeling [44,62,63,64].
- Changes in the deep capillary plexus are less pronounced but include alterations in perfusion and fractal parameters, particularly in central sectors [61,62,63].
- Peripapillary microcirculation is reduced and correlates with disease severity [44,64].

**Alzheimer’s Disease/Mild Cognitive Impairment:**

- In contrast, OCTA studies in AD and MCI demonstrate a consistent pattern of macular microvascular involvement. Most studies report significant reductions in VD in both superficial and deep macular plexuses, particularly in parafoveal and perifoveal regions, while foveal vascular changes, including enlargement of the FAZ, are evident in early or preclinical stages [48,65,66,67,68].
- Evidence from studies that evaluated vascular geometry further indicates decreased capillary complexity, reduced fractal dimension, fewer bifurcations, and lower vessel length density in AD and MCI, with MCI showing early alterations in both superficial and inner/deep vascular complexes [69,70].
- FAZ metrics are more variable but generally show enlargement, increased roundness, or greater tortuosity in MCI and preclinical AD, reflecting early microvascular remodeling prior to overt cognitive impairment [48,70].

In summary: Overall, neurodegenerative diseases such as PD and AD/MCI share microvascular alterations, including reduced VD and impaired capillary complexity, but exhibit distinct spatial patterns. In PD, these changes are regional and heterogeneous, with reductions in parafoveal and perifoveal VD, perfusion, and fractal dimension, while foveal regions may remain preserved or even show increased perfusion, potentially reflecting compensatory mechanisms [44,60,61,62,63,64]. By contrast, in AD and MCI, retinal microvascular involvement is more uniform, affecting both superficial and deep vascular complexes, with decreases in vessel length density, fractal dimension, and bifurcation number, alongside early remodeling of the FAZ [70]. Collectively, these findings highlight OCTA as a sensitive, non-invasive modality for the early detection and longitudinal monitoring of disease-specific microvascular alterations in neurodegenerative disorders.

### 4.3. Spectral-Domain Optical Coherence Tomography

SD-OCT, also called Fourier-domain or high-definition OCT, is an imaging technique based on low-coherence interferometry in which reflected light signals are captured and analyzed using a spectrometer, offering improved spatial resolution and acquisition speed compared to earlier OCT modalities. High-speed scanning reduces motion artifacts and enhances axial and transverse sampling, enabling detailed visualization of retinal microstructures [71].

These technical advantages allow SD-OCT to detect and characterize a wide range of structural alterations in the retina and choroid, including subtle morphological changes that occur during disease progression. As a result, SD-OCT has become a valuable tool for investigating microstructural retinal abnormalities associated with various pathological conditions [72,73].

#### SD-OCT Findings in PD and AD: Comparative Discussion

SD-OCT studies consistently demonstrate that both PD and AD involve structural alterations in the retina, particularly thinning of inner retinal layers. However, the patterns, timing, and biological implications of these changes differ between the two diseases.

**Parkinson’s Disease:**

In PD, SD-OCT reveals prominent thinning of the GCC, RNFL, and macular thickness. These changes correlate strongly with motor severity, disease duration, and age [74,75]. Cross-sectional studies confirm that RNFL and GCC thinning reflects neuroaxonal loss linked to dopaminergic dysfunction, while longitudinal data suggest limited predictive value for cognitive decline, indicating that retinal changes primarily mirror peripheral neurodegeneration rather than cortical pathology [76].

Similar patterns of thinning are observed in atypical parkinsonian syndromes and in conditions with dopaminergic involvement, reinforcing the notion that PD-associated retinal changes are closely tied to motor system degeneration [77,78].

**Alzheimer’s Disease:**

In contrast, AD shows a progressive, layer-specific retinal degeneration that aligns closely with cognitive impairment and cortical atrophy. SD-OCT consistently identifies thinning of the GCIPL and RNFL across the AD spectrum, from MCI to established dementia. Ibrahim et al. [79] demonstrated significant peripapillary RNFL thinning and inner and full retinal thickness reductions in both MCI and dementia groups compared with healthy controls, with stronger associations emerging after adjustment for confounders using ordinal logistic models. Importantly, RNFL thinning was linked to cognitive impairment severity rather than age alone.

Zhang et al. [80] confirmed these findings in early-onset AD, showing significant thinning of pRNFL, RNFL, and GCC compared to controls. Crucially, GCC thickness correlated with occipital lobe volume, providing structural brain–retina coupling and reinforcing the interpretation of retinal thinning as a marker of central neurodegeneration rather than ocular aging.

Wang et al. [81], using a large longitudinal UK Biobank cohort, extended these observations to the pre-diagnostic phase, showing that baseline GCIPL thinning predicted incident AD up to a decade later. Mediation analysis demonstrated that this association was partially driven by degeneration along the visual pathway, including cortical and subcortical gray matter loss and white matter disruption.

Preclinical stages may also involve outer retinal layers, including the photoreceptor inner segment, external limiting membrane, and RPE, which are associated with early vascular dysfunction and glial activation [82,83,84]. These findings suggest that retinal changes in AD begin years before clinical symptoms, reflecting amyloid- and tau-related neurodegeneration.

Although both PD and AD exhibit retinal structural abnormalities on SD-OCT, the pattern, timing, and biological interpretation of these changes differ substantially, reflecting distinct neurodegenerative mechanisms:

Layer specificity: AD exhibits early GCIPL involvement and outer retinal changes, whereas PD primarily affects RNFL and GCC.

Temporal profile: Retinal alterations in AD can precede cognitive symptoms, while in PD they parallel motor disease progression.

Underlying mechanisms: AD-related changes are linked to amyloid/tau pathology, glial activation, and neurovascular dysfunction, whereas PD changes are more closely associated with dopaminergic neurodegeneration and motor severity.

Overall, SD-OCT captures both shared and disease-specific retinal signatures. Inner retinal thinning is a general marker of neurodegeneration, while additional layer-specific patterns, temporal dynamics, and clinical correlations provide valuable clues for differentiating AD from PD. This highlights the potential of SD-OCT as a non-invasive tool for disease staging and as a complementary biomarker in the differential diagnosis of neurodegenerative disorders.

To provide a concise overview of retinal imaging alterations in AD and PD, Table 1 summarizes key findings across different modalities, including OCT, OCTA, and SD-OCT. The table highlights structural, vascular, and layer-specific changes, emphasizing both shared and disease-specific patterns. Inner retinal thinning, particularly of the RNFL and GCIPL, emerges as a common feature of neurodegeneration in both disorders, whereas outer retinal and RPE alterations are more characteristic of AD, often detectable in preclinical stages. Microvascular metrics, including VD and FAZ parameters, provide additional discrimination, with distinct spatial patterns observed in each disease. By synthesizing these findings, the table serves as a practical reference for understanding the comparative utility of retinal imaging biomarkers in early diagnosis, disease monitoring, and potential differential diagnosis of AD and PD.

To provide detailed study-level information including sample sizes, imaging methods, and reported performance metrics, Table 2 complements the summary in Table 1 by presenting key studies reporting retinal alterations in AD and PD using OCT-based imaging modalities.

Future Direction:

Future research should focus on large-scale, longitudinal studies using standardized OCT and OCTA protocols to clarify the temporal evolution of retinal changes in AD and PD. Integration of multimodal imaging approaches, including hyperspectral and functional retinal imaging, may enhance early detection and disease differentiation. Additionally, exploring the relationship between retinal biomarkers and molecular hallmarks of neurodegeneration, such as amyloid, tau, and α-synuclein, could strengthen their clinical relevance. Finally, the development of automated AI-based analysis tools and validation across diverse populations will be essential to translate retinal imaging biomarkers into routine clinical practice for early diagnosis and monitoring of neurodegenerative diseases.

## 5. Conclusions

This review highlights that the retina, owing to its unique embryological and functional continuity with the CNS, offers significant promise as a non-invasive surrogate for detecting and monitoring neurodegenerative changes associated with AD and PD. Advanced retinal imaging modalities such as OCT, OCTA, and SD-OCT consistently reveal structural and microvascular alterations in both disorders, reinforcing the concept that retinal biomarkers reflect broader cerebral pathology. Retinal thinning, particularly within the RNFL and GCIPL, and microvascular changes have been correlated with cognitive decline, brain atrophy, and disease severity, supporting their potential as accessible indicators of neurodegeneration.

Although retinal imaging biomarkers show considerable promise for early detection, differential diagnosis, and longitudinal monitoring, current evidence is limited by heterogeneous study protocols, inconsistent imaging standards, and varying cohort sizes. Further largescale, longitudinal studies with standardized imaging protocols are needed to validate and harmonize candidate biomarkers for translation into clinical practice.

In summary, retinal imaging represents a cost-effective and high-resolution approach with the potential to transform the early diagnosis and management of neurodegenerative diseases. Its integration into multimodal diagnostic frameworks may improve disease stratification and enable earlier therapeutic intervention, ultimately enhancing patient outcomes.

## Figures and Tables

**Table 1:** Retinal imaging findings in AD and PD.

Imaging Modality	Retinal Alterations	Alzheimer’s Disease	Parkinson’s Disease	Clinical Relevance
OCT	Inner retinal layers	Thinning of RNFL, GCIPL, and GCC; preferential temporal and inferior pRNFL involvement; heterogeneous across disease stages [47,48,49,50,51,52,53]	Predominant thinning of pRNFL, GCL, GCIPL, and GCC; relatively uniform pattern [34,35,36,37,38,39,41,42,43,44,45,46]	Reflects retinal neurodegeneration; correlates with cognitive decline in AD and motor severity in PD
OCT	Outer retinal layers	Alterations in outer retinal layers and RPE, particularly in preclinical and early AD [47,82,83,84]	Limited or inconsistent involvement of outer retinal layers; ONL may show thinning [34,38,45,46]	Highlights disease-specific retinal signatures; outer retinal changes more pronounced in AD than PD
OCTA	Macular microvasculature	Reduced VD and capillary complexity in both superficial and deep plexuses; early FAZ enlargement [48,65,66,67,68,69,70]	Reduced VD, perfusion, and fractal dimension mainly in parafoveal and perifoveal regions; foveal sparing or compensatory perfusion [44,60,61,62,63,64]	Microvascular biomarkers for early detection and disease monitoring
OCTA	FAZ metrics	Enlarged FAZ area, altered circularity and tortuosity, especially in MCI and preclinical AD [48,70]	Smaller FAZ area with increased complexity or lacunarity [44,62,63,64]	May help distinguish disease-specific microvascular remodeling
SD-OCT	Layer-specific thinning	Early and progressive GCIPL and RNFL thinning; strong association with cortical atrophy and cognitive decline [79,80,81]	RNFL and GCC thinning correlated with disease duration and motor severity [74,75,76,77,78]	Non-invasive biomarker for disease staging
SD-OCT	Temporal dynamics	Retinal changes detectable in preclinical and prodromal stages [47,49,50,82,83,84]	Retinal changes parallel established disease progression [74,75,76]	Supports early diagnosis in AD and monitoring in PD
SD-OCT	Pathophysiology	Amyloid- and tau-related neurodegeneration, glial activation, and neurovascular dysfunction [82,83,84]	Dopaminergic dysfunction and neuroaxonal loss [74,75,76,77,78]	Provides mechanistic insight into disease-specific pathology

**Table 2:** Summary of OCT-Based Studies on Retinal Changes in AD and PD: Methods, Sample Size, and Diagnostic Metrics.

Study	Sample Size	Method (Manual/AI)	Diagnostic Performance/Metrics
Salobrar-García et al., 2019, [28]	39 mild AD, 21 moderate AD, 40 controls	OCT/OCTA, Manual	Not reported
Lemmens et al., 2020, [31]	17 AD patients, 22 controls	OCT + Hyperspectral imaging; AI (linear discriminant classification)	AUC overall 0.74; RNFL features AUC 0.70–0.79
Murueta-Goyena et al., 2025, [40]	53 PD patients, 52 controls	SD-OCT, manual	Not reported
Zhang et al., 2021, [36]	78 PD patients (non-dementia)	OCT, manual	Not reported
Zhao et al., 2022, [35]	30 PD patients, 20 controls	OCT, manual	Not reported
Kamata et al., 2022, [37]	14 PD patients, 22 controls	OCT, manual	Not reported
Tran et al., 2024, [38]	16 PD patients, 21 controls	OCT, manual + electroretinography	Not reported
Tu et al., 2023, [39]	56 PD patients, 45 controls	OCT/OCTA, manual	Not reported
Elanwar et al., 2023, [41]	50 PD patients, 50 controls	OCT, manual + full-field electroretinogram	Not reported
Christou et al., 2023, [44]	32 PD patients, 46 controls	OCT, manual	Not reported
Zhao et al., 2021, [46]	6 PD patients, 32 controls	SD-OCT with deep learning segmentation	Not reported
Gao et al., 2024, [47]	35 SCD, 36 cognitive impairment, 29 normal cognition	OCT/OCTA, manual	Not reported
O’Bryhim et al., 2018, [48]	14 preclinical AD, 16 controls	OCTA automated software measurements	FAZ AUC = 0.8007 (95% CI 0.6647–0.9367)
Chua et al., 2022, [49]	62 AD patients, 108 MCI patients, 55 controls	Automated OCT analysis	cpRNFL measured: AUC 0.69; macular layers (mRNFL + mGCL + mIPL): AUC 0.73; cpRNFL compensated: AUC 0.74; Combined macular + cpRNFL compensated: AUC 0.80
Mathew et al., 2023, [50]	75 participants (28 cognitively normal, 26 SCD, 17 MCI, 4 AD)	Manual OCT measurement	Not reported
Shi et al., 2020, [61]	25 PD patients, 25 healthy controls	OCTA, automatic segmentation	Not reported
Murueta-Goyena et al., 2021, [62]	55 PD patients, 48 controls	OCTA, manual	Not reported
Eker et al., 2025, [63]	41 PD patients, 41 healthy controls	OCTA, automatic segmentation	Not reported
Xu et al., 2022, [64]	115 PD patients, 67 healthy controls	OCTA, manual	Not reported
Lahme et al., 2018, [65]	36 AD patients, 38 healthy controls	OCTA, automatic segmentation	Not reported
Yoon et al., 2019, [66]	39 AD patients, 37 MCI patients, 133 controls	OCTA, automatic segmentation	Not reported
Zabel et al., 2019, [67]	27 patients with AD, 27 healthy controls	OCTA, manual	Not reported
Chua et al., 2020, [68]	24 AD patients, 37 MCI patients, 29 controls	OCTA, automatic segmentation	Superficial fractal dimension (AUC = 0.77), superficial vessel density (AUC = 0.72), deep vessel density (AUC = 0.64); MRI medial temporal atrophy score AUC = 0.56
Wu et al., 2020, [69]	18 AD patients, 21 MCI patients, 21 healthy controls	OCTA	Not reported
Xie et al., 2024, [70]	55 AD, 41 MCI, 62 healthy controls	OCTA with deep learning–based segmentation	Not reported

## Data Availability

No new data were created or analyzed in this study. Data sharing is not applicable to this article.

## Abbreviations

The following abbreviations are used in this manuscript:

AD	Alzheimer’s disease
PD	Parkinson’s disease
CNS	Central nervous system
OCT	Optical Coherence Tomography
OCTA	Optical Coherence Tomography Angiography
SD-OCT	Spectral-Domain Optical Coherence Tomography
RNFL	Retinal nerve fiber layer
pRNFL	Peripapillary retinal nerve fiber layer
GCL	Ganglion cell layer
IPL	Inner plexiform layer
GCIPL	Ganglion cell–inner plexiform layer
GCC	Ganglion cell complex
INL	Inner nuclear layer
RPE	Retinal pigment epithelium
SCP	Superficial capillary plexus
VD	Vessel density
FAZ	Foveal avascular zone
MCI	Mild cognitive impairment
SCD	Subjective cognitive decline
